# Supplementary material for: Histone H2AX deficiency causes neurobehavioral deficits and impaired redox homeostasis
Source: Nat Commun. 2018 Apr 18;9:1526. doi: 10.1038/s41467-018-03948-9 (PMC5906610; doi:10.1038/s41467-018-03948-9)

Supplementary Information

Histone H2AX deficiency causes neurobehavioral deficits and impaired redox homeostasis

Weyemi Urbain, Bindu D. Paul, Adele M. Snowman, Parthav Jailwala, Andre Nussenzweig, William M. Bonner, and Solomon H. Snyder.

Supplementary Figures

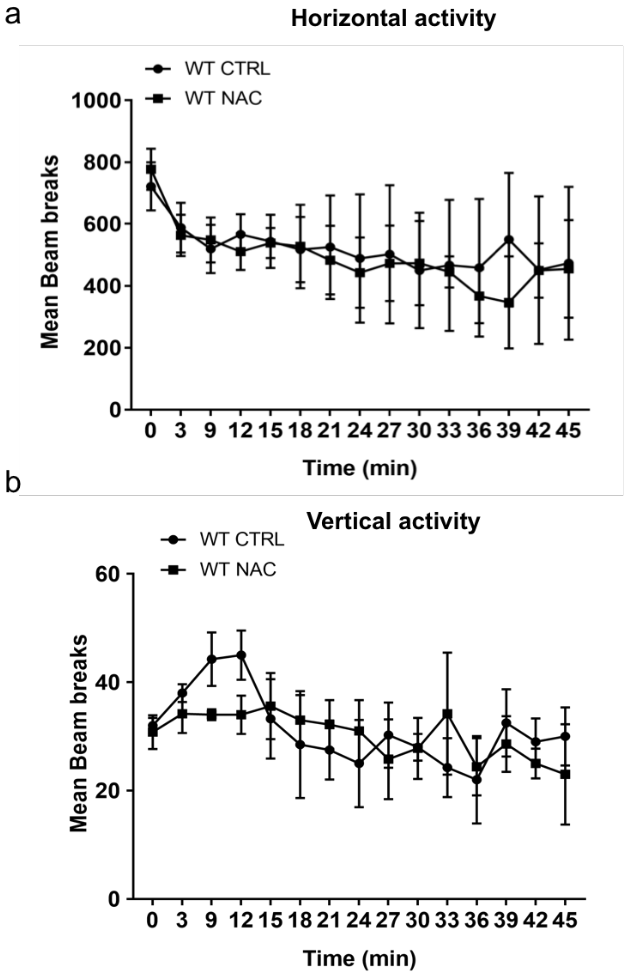

Supplementary Figure 1: NAC treatment does not affect the general locomotor activity of wild type mice. Open-field testing demonstrated that N-acetyl-cysteine (NAC 20 mM) has no effect on the general locomotor activity of H2AX wild type mice as measured by horizontal activity (**a**), or vertical activity (**b**); y-axis, beam breaks, x-axis, 3 min intervals. Data were recorded at the end of NAC treatment (4 months)**;** n=5 (means ± s.e.m.) for H2AX wild type control group; n=5 (means ± s.e.m.) for H2AX wild type treated with NAC.

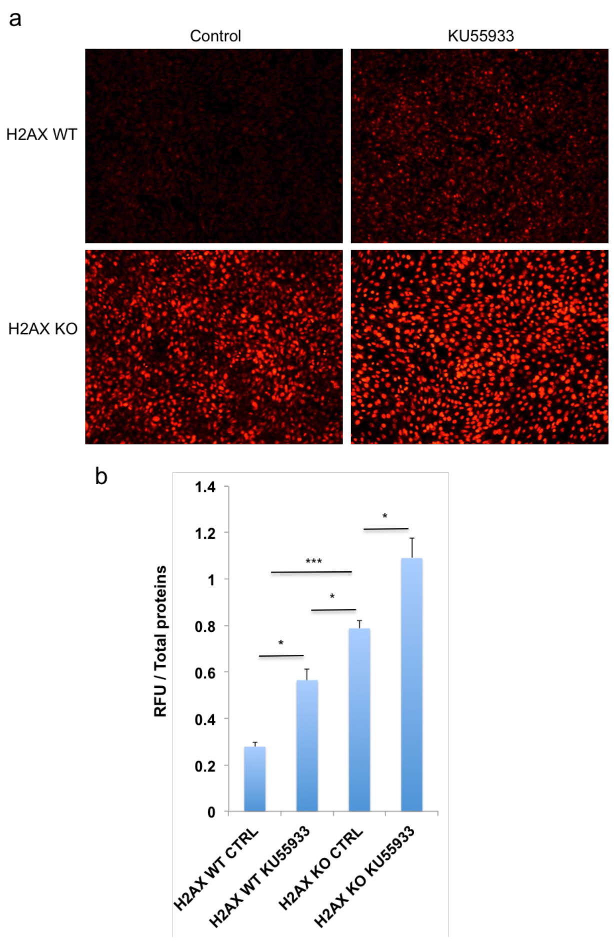

Supplementary Figure 2: Treatment of H2AX knockout cells with ATM specific inhibitor (KU55933) results in accrued ROS levels. **(a)** Inhibition of ATM activity was performed using treatment with KU55933. Cells were treated with 10 μM of KU55933 for 24 hrs and stained with dihydroethidium (DHE). Control cells (H2AX WT) as well as mutant mouse embryonic fibroblasts (H2AX KO) treated with KU55933 or with DMSO (control), were incubated with DHE and used for ROS detection using microscopy. **(b)** Fluorescence intensity was quantified using a Plate Reader, and data are means ± s.d.; n = 3. Statistical significance was determined by a two-tailed, unpaired Student's *t*-test, *P < 0.05, ***P < 0.0001.

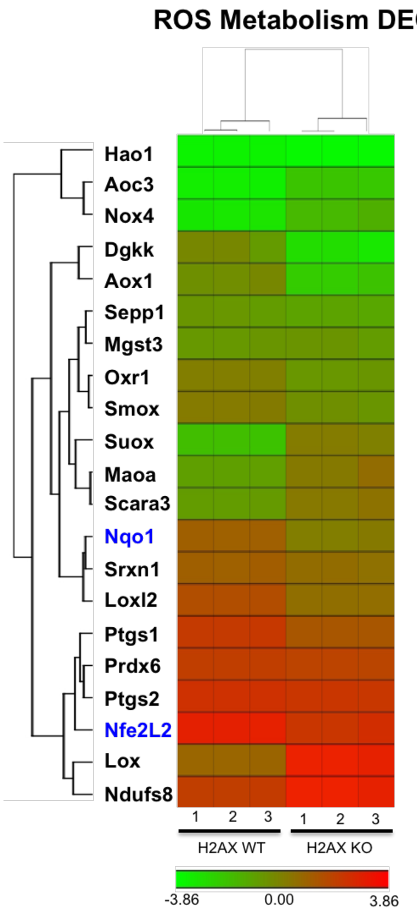

Supplementary Figure 3: H2AX deletion promotes significant changes in the expression of ROS metabolism genes. The Differential gene expression analysis comparing H2AX-deficient and control mouse embryonic fibroblasts was performed. A heat map was generated by creating a list of genes involved in reactive oxygen species (ROS) metabolism from the 1295 differentially expressed genes (DEGs), on the basis of their annotation in Gene Ontology. Agglomerative hierarchical clustering was carried out in Partek using average linkage method and Euclidean dissimilarity measure for both rows and columns. The heat map of the 21 Differentially Expressed Genes (DEGs) involved in ROS metabolism was generated. *NRF2* (*Nfe2L2*) and its major transcriptional target *NQO1* are significantly downregulated in H2AX mutant cells. Additional targets include *SRXN1*, *PTGS1*, *PTGS2* and *PRDX6*. The numbering refers to independent replicates for either control cells (WT) or H2AX mutant cells (KO).

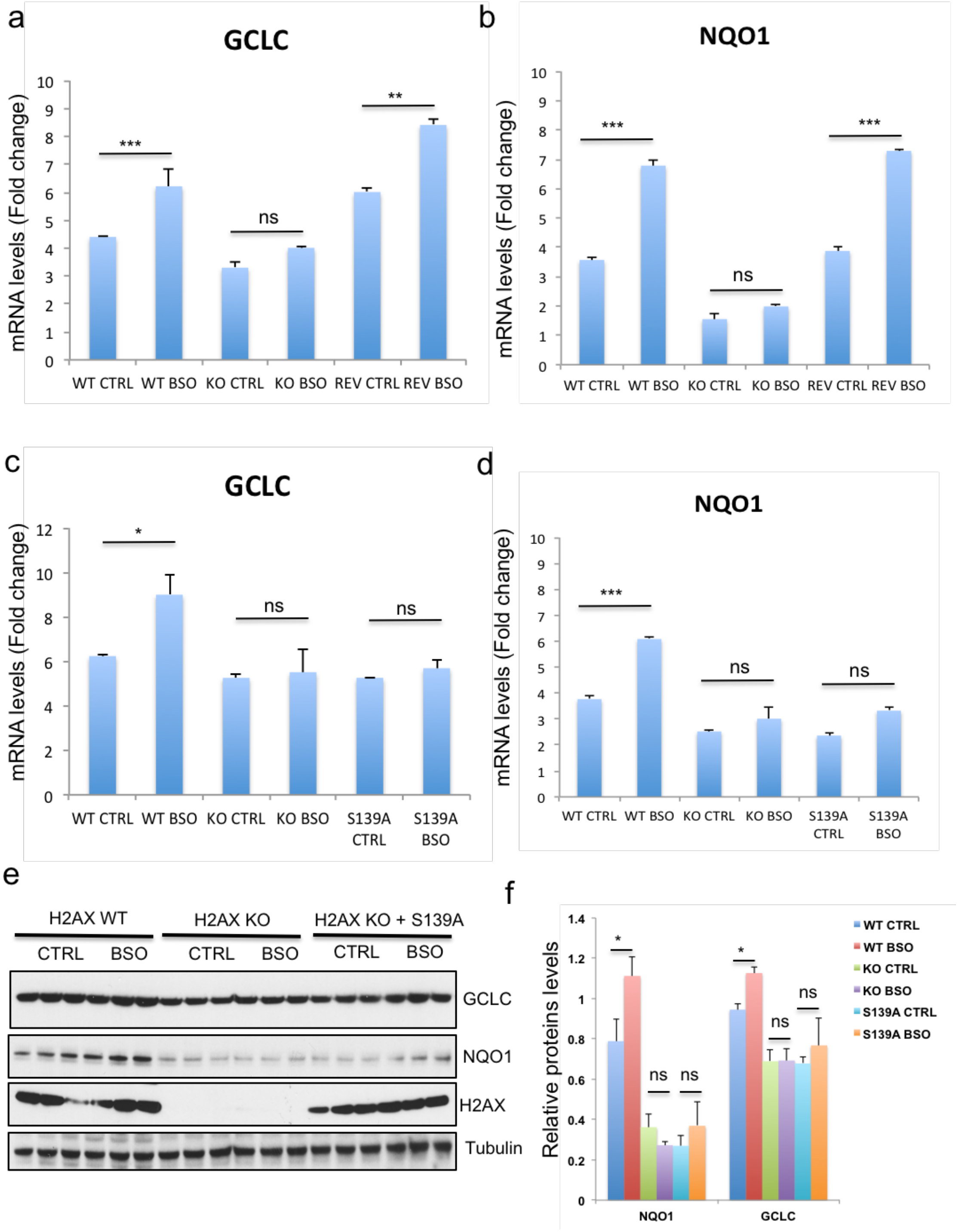

Supplementary Figure 4: Ectopic expression of H2AX mutant (S139A) fails to restore NRF2-transcriptional targets GCLC and NQO1 in response to oxidative stress. (a,b) Analysis of NQO1 and GCLC transcript levels by real time PCR in parental cells (WT), H2AX knockout cells (KO) and H2AX

knockout cells in which H2AX expression was restored (REV: revertants). Cells were treated for 24 hrs with 2 mM of BSO to promote endogenous oxidative stress. Expression values are relative fold change for gene transcripts normalized to GAPDH. Error bars represent s.e.m (n=3), **P < 0.001, ***P < 0.0001, ns, non-significant (P = 0.0636 for GCLC and 0.0763 for NQO1). **(c,d)** BSO treatment results in impaired activation of NQO1 and GCLC transcripts in in H2AX knockout cells (KO), as well as in H2AX knockout cells in which an ectopic expression of a mutant H2AX (S139A) was performed. Analysis of NQO1 and GCLC transcript levels was performed by real time PCR. Expression values are relative fold change for gene transcripts normalized to GAPDH. Error bars represent s.e.m (n=3), *P < 0.05, ***P < 0.0001, ns, non-significant (P = 0.8217 and 0.6738 for GCLC; and P = 0.3469 and 0.2031 for NQO1). **(e,f)** Immunoblot analysis of GCLC, NQO1 in parental cells (WT), in H2AX knockout cells (KO), as well as in H2AX knockout cells in which an ectopic expression of a mutant H2AX (S139A) was performed. Cells were treated for 24 hrs with 2 mM buthionine sulfoximine (BSO) to induce endogenous oxidative stress. **(e),** representative image, **(f)**, quantification. Error bars represent the s.e.m. (*n*=3). Statistical significance was determined by a two-tailed, unpaired Student's *t*-test. *P < 0.05, ns, non-significant (P = 0.0780 and 0.2535 for NQO1; and P = 0.9404 and 0.3181 for GCLC).

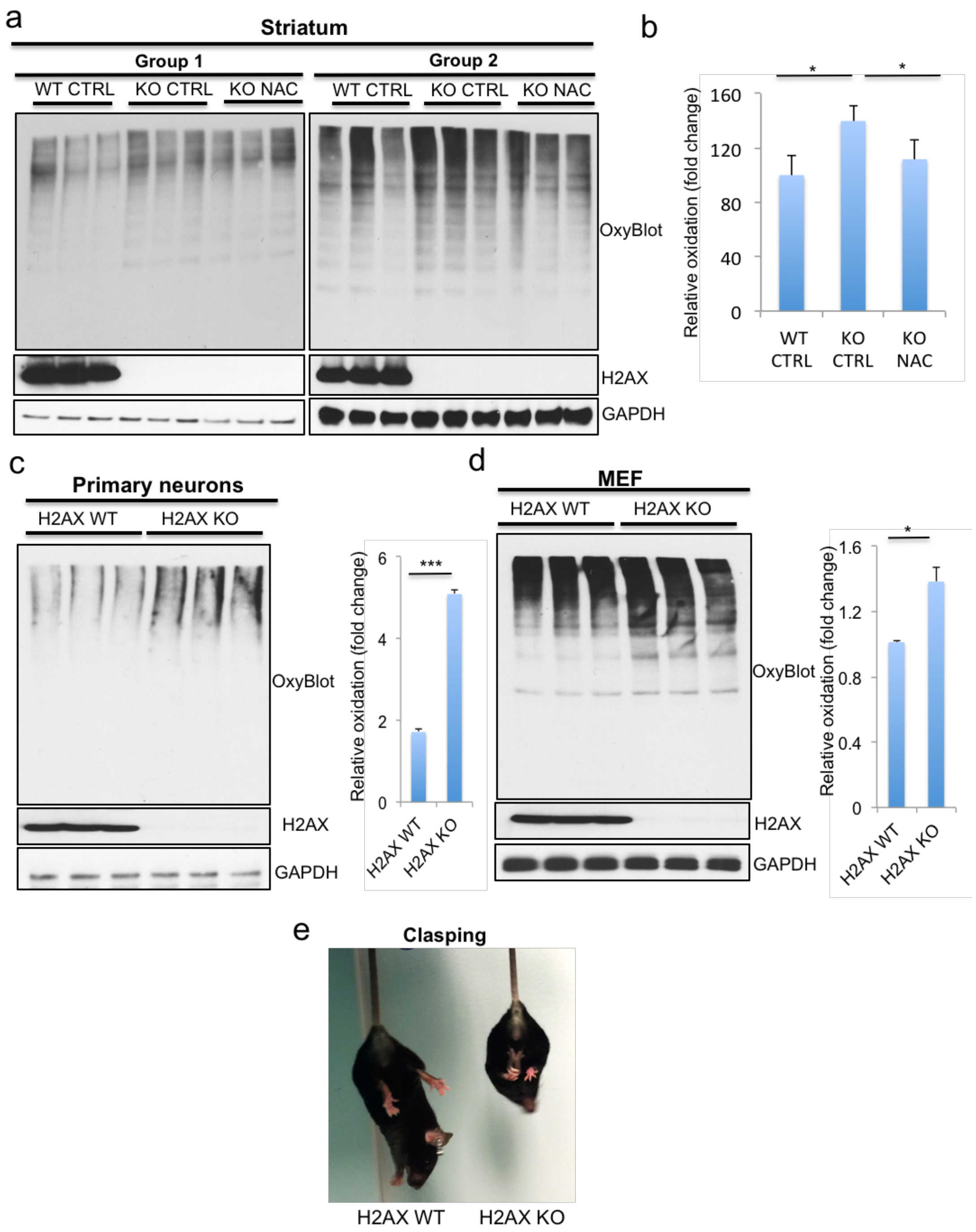

Supplementary Figure 5: H2AX deletion induces elevated protein oxidation in the brain. (a) Western blot analysis of protein carbonylation (OxyBlot™). H2AX knockout mice received N-acetyl-cysteine (NAC 20 mM) in their drinking water from the weaning up to 4 months. Protein lysates from the striatum were used to assess the global protein oxidation using the OxyBlot kit, as described above. **(b)** The

density of the bands shown in **(a)** was quantified using *Image J.* Error bars represent the s.e.m. (*n*=6). Statistical significance was determined by a two-tailed, unpaired Student's *t*-test. *P < 0.05. **(c,d)** Western blot analysis of the levels of protein carbonylation (OxyBlot) in primary cortical neurons from both wild-type and H2AX knockout mice **(c)**; and in mouse embryonic fibroblasts **(d)**. The density of the bands in the left panels was quantified using *Image J*, and is shown in the right panels. Error bars represent the s.e.m. (*n*=3). Statistical significance was determined by a two-tailed, unpaired Student's *t*-test. *P < 0.05, ***P < 0.0001. **(e)** H2AX knockout mice show a limb-clasping phenotype, reminiscent of mouse models with oxidative lesions in brain regions such as the striatum. The limb-clasping phenotype was monitored, by suspending both wild type and H2AX knockout mice by their tail.

Gene	WT1	WT2	WT3	KO1	KO2	KO3	Gene assignment	Refseq	p-value(KO vs. WT)	Fold change (KO vs Wt)	Fold change direction
Aox1	7.95816	8.04442	8.19984	5.90982	5.51737	5.51737	NM_009676 // Aox1 //	NM_009676	0.000061259	-5.10056	KO down vs WT
Ptgs2	10.871	10.871	10.8441	10.1055	10.0552	10.0552	NM_011198 // Ptgs2 //	NM_011198	2.52178E-05	-1.66562	KO down vs WT
Prdx6	10.4662	10.4662	10.4535	9.67968	9.66086	9.66086	NM_001303408 // Prdx	NM_001303408	6.28676E-06	-1.70109	KO down vs WT
Mgst3	7.73838	7.73838	7.76255	7.05016	7.18881	7.18881	NM_025569 // Mgst3 /	NM_025569	0.000103607	-1.54492	KO down vs WT
Suox	6.57583	6.57583	6.42575	7.91919	7.9278	7.9278	NM_173733 // Suox //	NM_173733	1.56767E-05	2.71311	KO up vs WT
Aoc3	5.13915	5.13915	5.04451	5.71832	5.74803	5.74803	NM_009675 // Aoc3 //	NM_009675	0.0114477	1.68145	KO up vs WT
Loxl2	9.96504	9.96504	9.95023	8.48862	8.32839	8.32839	NM_033325 // Loxl2 //	NM_033325	0.00027385	-2.67578	KO down vs WT
Scara3	7.60871	7.60871	7.62882	8.32333	8.04784	8.04784	NM_172604 // Scara3 /	NM_172604	0.00457276	1.62003	KO up vs WT
Sepp1	7.78013	7.78013	7.58438	6.72981	6.71583	6.71583	NM_001042613 // Sepp	NM_001042613	0.000649604	-2.11192	KO down vs WT
Oxr1	8.51115	8.51115	8.45497	7.25635	7.1123	7.1123	NM_001130163 // Oxr1	NM_001130163	0.00017613	-2.29049	KO down vs WT
Lox	9.23557	9.23557	9.24902	11.0317	11.1103	11.1103	NM_001286181 // Lox /	NM_001286181	2.61438E-07	3.59917	KO up vs WT
Ndufs8	10.4616	10.4616	10.5034	11.0596	11.1819	11.1819	NM_001271443 // Ndu	NM_001271443	0.000289796	1.57283	KO up vs WT
Ptgs1	10.5786	10.5786	10.5973	9.17309	9.0507	9.0507	NM_008969 // Ptgs1 //	NM_008969	8.01757E-06	-2.7008	KO down vs WT
Smox	8.61892	8.61892	8.5557	7.23115	7.37811	7.37811	NM_001177833 // Smo	NM_001177833	6.58912E-05	-2.42915	KO down vs WT
Srxn1	9.44279	9.44279	9.48828	8.35734	8.41631	8.41631	NM_029688 // Srxn1 //	NM_029688	2.05322E-06	-2.13369	KO down vs WT
Hao1	4.96866	4.96866	4.89561	4.06979	3.97513	3.97513	NM_010403 // Hao1 //	NM_010403	0.00103959	-1.74685	KO down vs WT
Nox4	5.32757	5.32757	5.39799	6.46798	6.09464	6.09464	NM_001285833 // Nox	NM_001285833	0.00161787	1.90102	KO up vs WT
Dgkk	8.22537	8.22537	7.58762	4.74782	4.99849	4.99849	NM_177914 // Dgkk //	NM_177914	0.000107842	-8.17933	KO down vs WT
Maoa	7.51283	7.51283	7.52545	8.4975	8.03871	8.03871	NM_173740 // Maoa //	NM_173740	0.00536322	1.82158	KO up vs WT
Nfe2L2	11.5284	11.5284	11.5671	10.4251	10.0493	10.0493	NM_010902 // Nfe2l2 /	NM_010902	0.000358145	-2.41806	KO down vs WT
Nqo1	9.44264	9.44264	9.41133	8.11191	7.87799	7.87799	NM_008706 // Nqo1 //	NM_008706	8.28384E-05	-2.64686	KO down vs WT

Supplementary Table 1: List of the genes shown in the heat map summarizing differential expression of genes involved in ROS metabolism. Genome-wide expression analysis comparing H2AX-deficient and control mouse embryonic fibroblasts was performed. The list of genes involved in ROS metabolism was created using their annotation in Gene Ontology.

Supplementary Note 1

Protein carbonyl assay (OxyBlot™ analyses)

Protein carbonyls were assayed by Western blot analysis in mouse embryonic fibroblasts (MEFs), primary cortical neurons and the striatum from both wild type and H2AX knockout mice, according to the manufacturer's instructions (OxyBlot™ Protein Oxidation Detection Kit (S7150); Millipore Corporation, USA). Briefly, 15 µg proteins were reacted with 2,4 dinitrophenylhydrazine and western blotted using a primary antibody specific to dinitrophenylhydrazone-derivatized residues (OxyBlot™) and a secondary antibody (OxyBlot™). Protein carbonyls were visualized by enhanced chemioluminescence and quantified by densitometry. The density of the bands was quantified using *Image J* (Java-based image processing program developed at the National Institutes of Health). GAPDH was used as a loading/internal control.

Uncropped immunoblot images

Fig. 3c

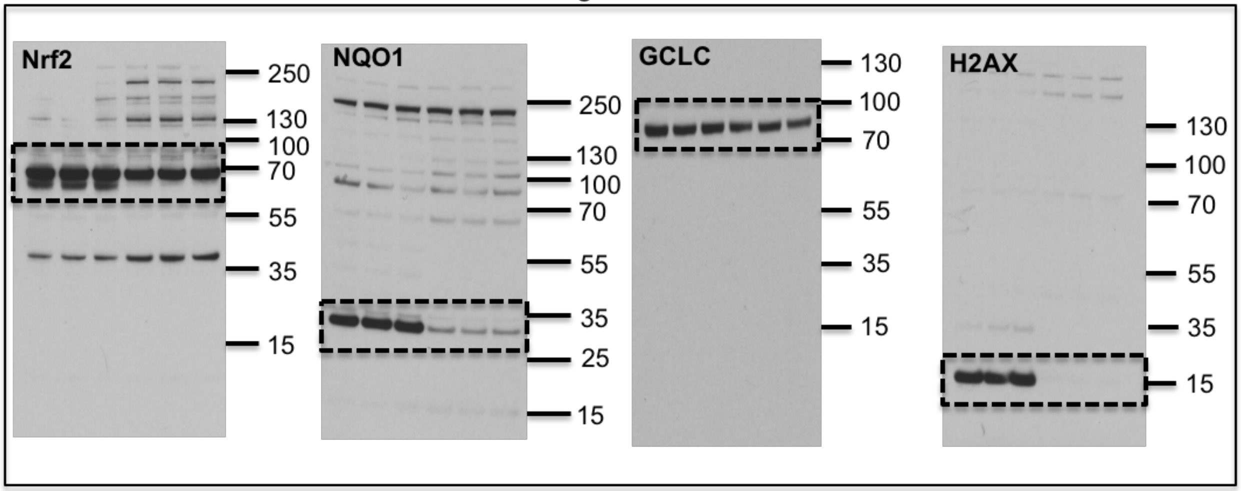

Fig. 4a

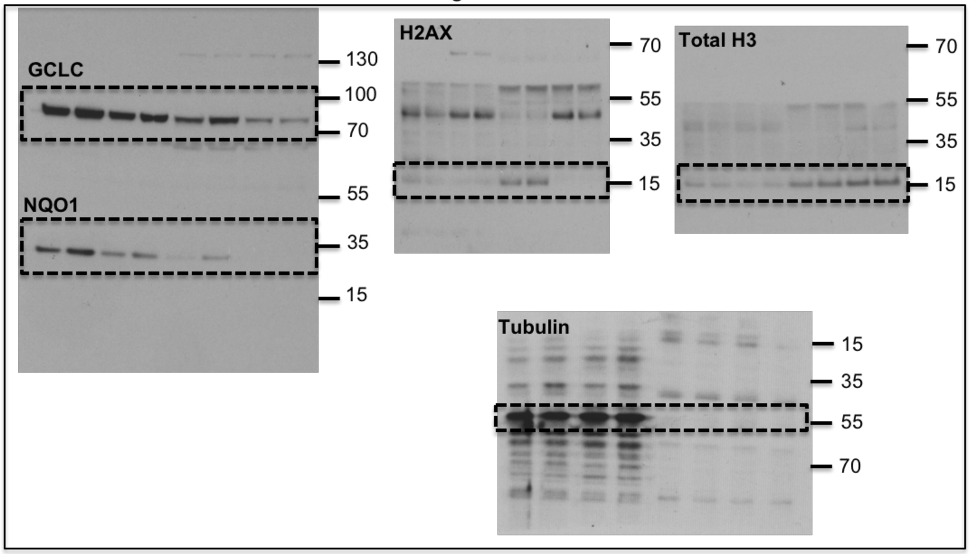

Fig. 4c

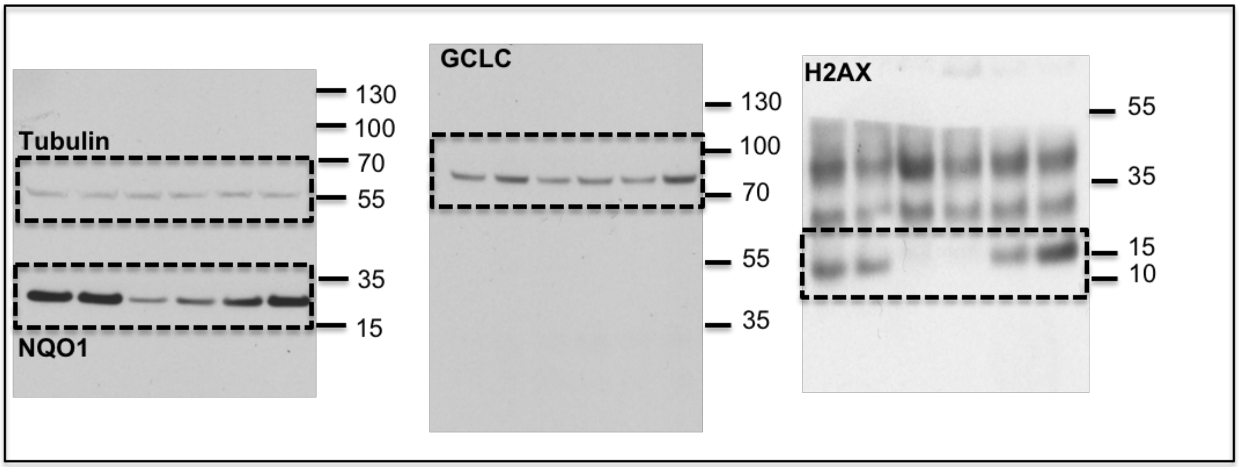

Fig. 4e

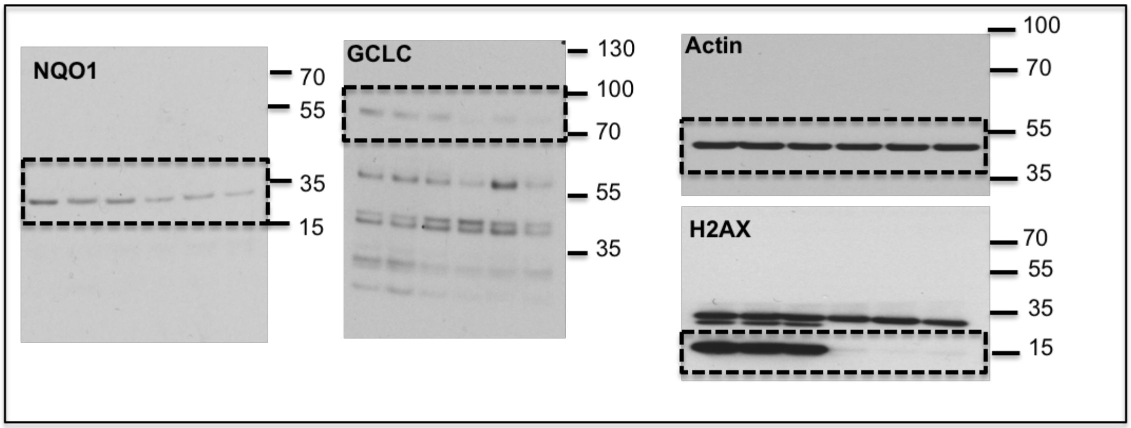

Supplementary Figure 4e

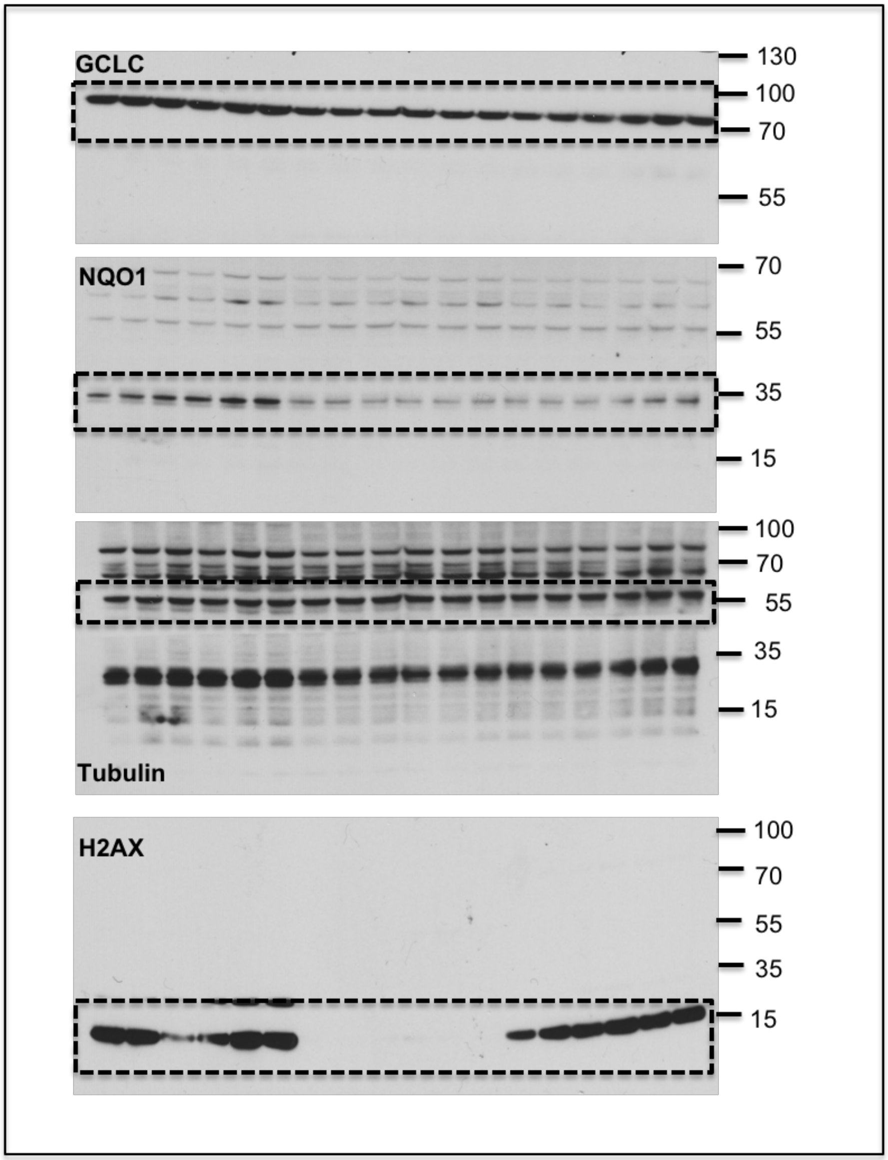

Supplement: Supplementary file 1 — Supplementary Information [file 41467_2018_3948_MOESM1_ESM.pdf]
